# Potential new therapeutic modality revealed through agent-based modeling of the neuromuscular junction and acetylcholinesterase inhibition

**DOI:** 10.1186/1742-4682-11-42

**Published:** 2014-10-02

**Authors:** Richard R Chapleau, Peter J Robinson, John J Schlager, Jeffery M Gearhart

**Affiliations:** Henry M Jackson Foundation for the Advancement of Military Medicine, 2729 R Street, Wright Patterson AFB, OH 45433 USA; Molecular Bioeffects Branch, Bioeffects Division, 711th Human Performance Wing, Human Effectiveness Directorate, Air Force Research Laboratory (711 HPW/RHDJ), 2729 R Street, Wright Patterson AFB, OH 45433 USA

**Keywords:** Neuromuscular junction, Acetylcholinesterase, Organophosphate intoxication, Medical countermeasure, Allosteric enzyme regulation, Agent-based model

## Abstract

**Background:**

One of the leading causes of death and illness within the agriculture industry is through unintentionally ingesting or inhaling organophosphate pesticides. OP intoxication directly inhibits acetylcholinesterase, resulting in an excitatory signaling cascade leading to fasciculation, loss of control of bodily fluids, and seizures.

**Methods:**

Our model was developed using a discrete, rules-based modeling approach in NetLogo. This model includes acetylcholinesterase, the nicotinic acetylcholine receptor responsible for signal transduction, a single release of acetylcholine, organophosphate inhibitors, and a theoretical novel medical countermeasure. We have parameterized the system considering the molecular reaction rate constants in an agent-based approach, as opposed to apparent macroscopic rates used in differential equation models.

**Results:**

Our model demonstrates how the cholinergic crisis can be mitigated by therapeutic intervention with an acetylcholinesterase activator. Our model predicts signal rise rates and half-lives consistent with *in vitro* and *in vivo* data in the absence and presence of inhibitors. It also predicts the efficacy of theoretical countermeasures acting through three mechanisms: increasing catalytic turnover of acetylcholine, increasing acetylcholine binding affinity to the enzyme, and decreasing binding rates of inhibitors.

**Conclusion:**

We present a model of the neuromuscular junction confirming observed acetylcholine signaling data and suggesting that developing a countermeasure capable of reducing inhibitor binding, and not activator concentration, is the most important parameter for reducing organophosphate (OP) intoxication.

**Electronic supplementary material:**

The online version of this article (doi:10.1186/1742-4682-11-42) contains supplementary material, which is available to authorized users.

## Background

Inadvertent or intentional ingestion of organophosphate (OP) pesticide is a common occurrence in agricultural areas [1] and OP nerve agents remain a threat in chemical terrorism [2]. Efforts to develop new therapeutic treatments for OP poisonings are frequently resulting in new oxime-based reactivators [3], stoichiometric bioscavengers [4], or catalytic scavengers [5]. These treatments rely upon knowledge and identification of an acute exposure, must be administered within a narrow therapeutic window, and are not broad treatments but are selective for specific OPs. Despite these limitations, the currently deployed OP therapeutics used in the U.S. (the Mark 1 pralidoxime/atropine autoinjector and pyridostigmine bromide) have been in use since the 1950s.

Current advanced computational models of OP intoxication are constructed as physiologically-based pharmacokinetic (PBPK) models in order to estimate target tissue dosimetry. These models are then connected to pharmacodynamic (PD) models to predict the biological response at the target site. Such models exist for paraoxon [6, 7], diazinon [8, 9], chlorpyrifos [9], diisopropylfluorophosphate (DFP) [7, 10], and more recently for soman [11]. The model for paraoxon poisoning was developed for the rainbow trout while the models for diazinon and DFP, in contrast, were validated against rat and human *in vivo* data. More broadly applicable models were developed for soman [11] and for dermal absorption of pesticides such as parathion and fenthion [12]. The primary advantage of these PBPK models is that they can provide an accurate estimate of population behaviors and predict systemic outcomes.

**Figure 1:**
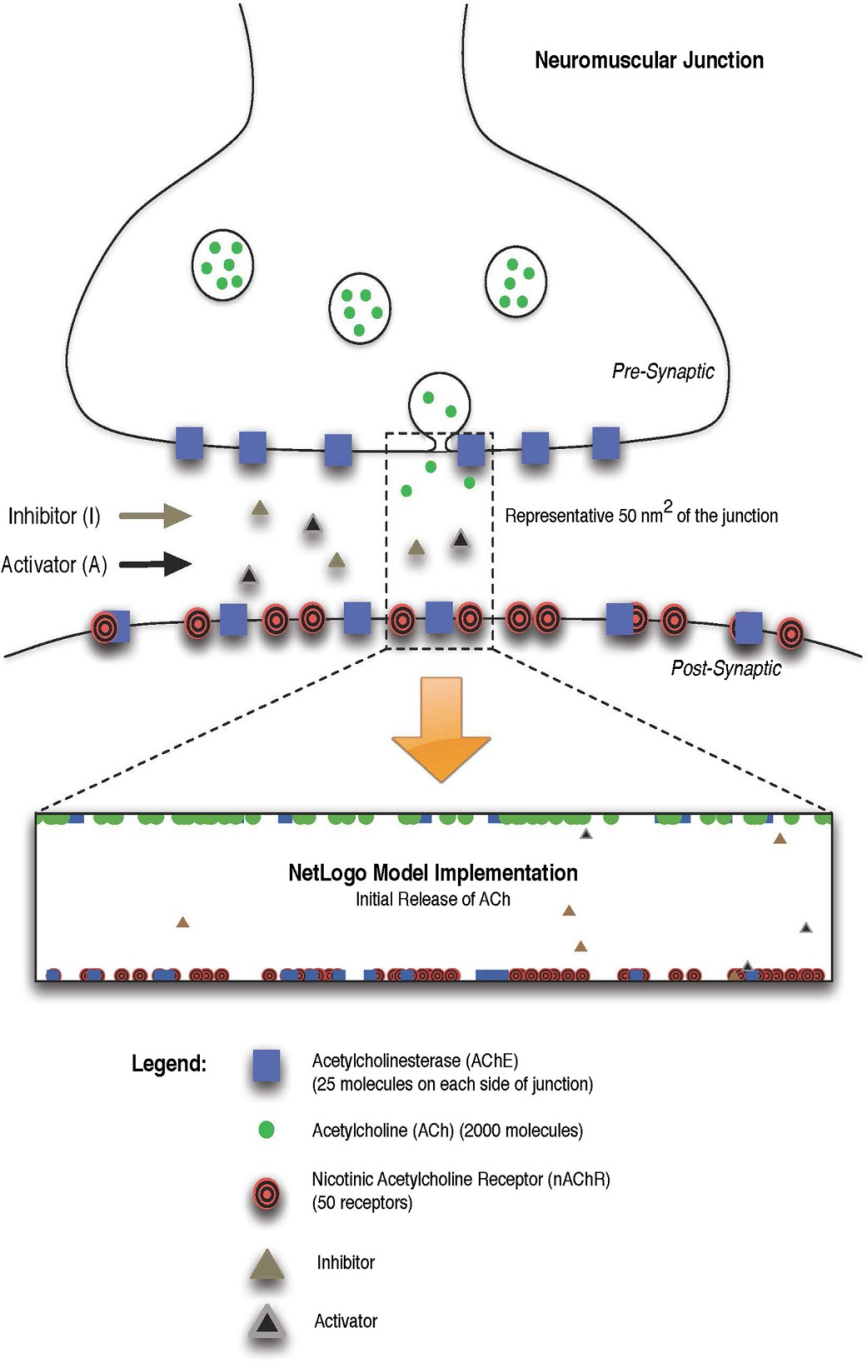
**Conceptual rendering of the neuromuscular junction and NetLogo rendering of the junction as relates to the model.**

The work presented here develops a PD model of the mammalian neuromuscular junction (NMJ) based on an agent-based model (ABM) describing acetylcholine signaling through nicotinic receptors (Figure 1). Agent-based modeling is a discrete, rules-based method of computational modeling that focuses on the individual components of an experimental system to perform *in silico* experiments [13]. ABMs are well suited for cases where the modeling goal is to test the validity of a mechanistic hypothesis [14], such as the case herein where allosteric activation of AChE is proposed to protect against OP intoxication. For example, the use of an ABM to model signaling events in the NF-κB pathway showed strong correlation between ABM, differential equation approaches (ODE), and *in vitro* measurements [15]. Lipniacki [16] showed that a purely ODE approach within the NF-κB system does not fully account for singular events, which required superposition of stochastic modeling onto the ODE. Furthermore, a recent modeling method has extended the ABM to include even finer resolution of physical space in chemical reactions, generating a spatial model of toll-like receptor 4 immune signaling that qualitatively reproduced the observed dynamics of tumor necrosis factor secretion [17].

Compared with ODEs, ABM constructs are readily adapted to spatial dimensions [18]; are stochastic by nature; can easily incorporate new information by adding more agent-types or modifying rules without rewriting the entire simulation; and reproduce emergent behaviors through parallelism and stochasticity [14]. Models in the ABM paradigm can be assembled even when complete knowledge of the system being simulated is lacking, as, for example, in the case herein where the characteristics of an enzyme activator are theoretical. Finally, ABMs describe the behavior of individuals such that the simulation does not always follow the average behavior that the ODE description would provide, thus taking into account the often significant impact of “outlier events” on the overall biological process. Although the system outcome from each ABM run is different, multiple runs provide a non-parametric means to explore the variability of outcomes, including the impact of rare events, eventually converging with the ODE-based results.

Traditional ODE models can be successfully employed to predict macroscopic effects that are changing in a continual manner; however they fall short in modeling dynamic processes such as biological systems that can change properties over time [19]. The NMJ modeled here is a particularly unique example of a dynamic biological system. The model includes a single release of acetylcholine (2000 molecules) from the neuron into a 50 nm^2^ region of the junction, containing 25 acetylcholinesterase molecules (biologically, these are tetramers treated individually) on each side of the junction and 50 nicotinic acetylcholine receptors (nAChR). When an individual acetylcholine molecule interacts with either the enzyme or the receptor, the agents both change.

## Results and discussion

The model described here permits small molecular agents (i.e. ACh, inhibitor, and activator) to travel through the neuromuscular junction and interact with proteins (i.e. AChE or nAChR), binding and dissociating according to their state. Each agent is a biological entity and the interactions between protein and small molecule are based upon experimentally determined rate constants. As with the real-world, this model is limited in that interactions can only occur when two criteria are satisfied: two agents must be in physical proximity to each other, and they each must be capable of binding (i.e. no partner for ACh, and 0 or 1 partner for nAChR). To maintain consistency with the reality of a single endosome release, the model is spatially constrained along the y-axis to the junction distance, while the x-axis is allowed to remain unconstrained to simulate diffusion into and out of the region of interest.

**Figure 2:**
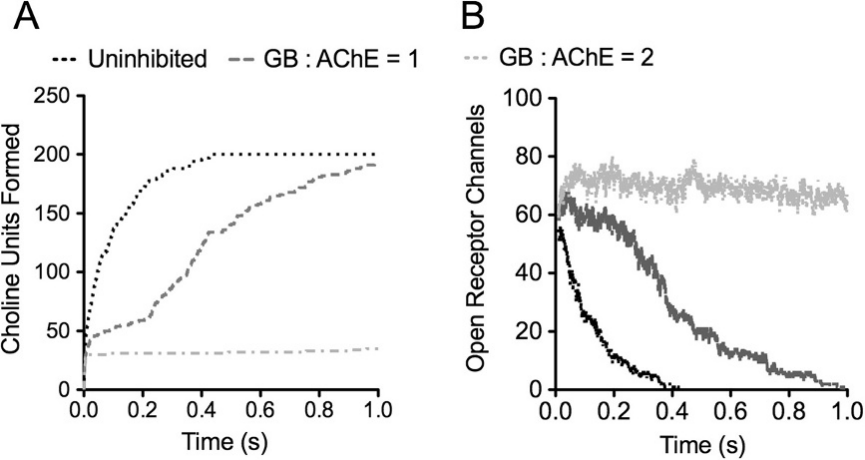
**Model validation with active and GB inhibited AChE at two GB:AChE ratios (1, 2).** Rates of ACh turnover are consistent with published values **(A)** and inhibition elongates the e.p.p. **(B)**. Legend refers to both A and B.

To measure model performance and ensure the accuracy of the predictions generated, a set of simulations were performed wherein neither inhibitors nor activators were present and also with full AChE inhibition (Figure 2). The predicted shape and response to AChE inhibition is in agreement with *in vitro* observations [20, 21], and the predicted end-plate potential (e.p.p.; represented by number of open receptor channels in our model) rise-time of 35 msec and half-life of 94 msec are comparable with observed values of 6 msec and 7 msec, respectively [20]. The nearly 10-fold discrepancy between prediction and observation is not unexpected, as these references provide data from an amphibian leg muscle measurement and also demonstrated that both the e.p.p. rise-time and half-life can be affected by extracellular ionic strength, membrane potential, amount of ACh released, and the placement of electrodes for measurements. Previous *ex vivo* data in frog muscles show similar time scales and half-lives in the presence and absence of AChE inhibitors [21].

Inhibition of AChE activity sharply decreases ACh clearance and elongates the e.p.p. (Figure 2). When maximally inhibited by GB (GB to AChE ratio of 2), the e.p.p. rise time was still 35 msec, however the half-life increased to 5.8 s. Inhibition at an inhibitor-enzyme ratio of 1 resulted in the appearance of two ACh turnover phases, the fast phase corresponding to the first 220 msec with a plateau equivalent to the fully inhibited level and a half-life of 2.3 msec, and a second, slower phase with a half-life of 211 msec. The e.p.p. prediction (Figure 2B) in this case also exhibited a two state response, with a signal plateau of 209 msec before signal decay with a half-life of 206 msec. Clearly, AChE inhibition affects multiple aspects of ACh clearance and the resulting e.p.p. duration.

**Figure 3:**
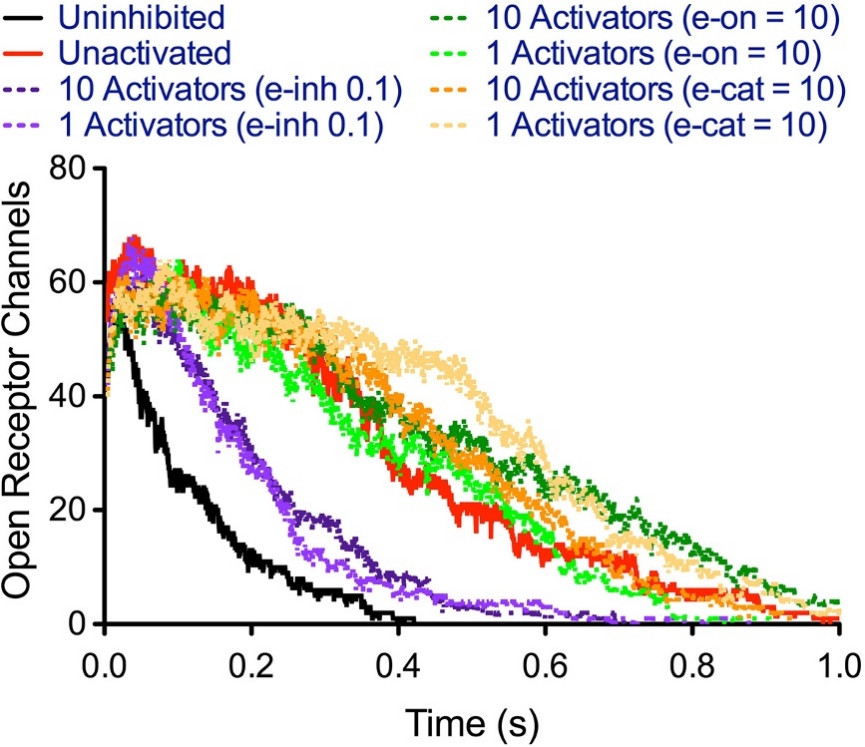
**Prediction of effects of activating AChE on e.p.p. duration in the presence of a 1:1 GB:AChE ratio.** Varying activation parameters has a greater effect than varying activator concentration.

To test the effects of general activation as a therapy for AChE inhibition by OPs, the impacts of small changes in activation parameters and activator concentrations on the e.p.p. were evaluated (Figure 3). By altering these parameters by 10, the inhibitor binding coefficient (*ε-inh*), was identified as the most critical parameter in affecting e.p.p. duration. Following the demonstration that modulating inhibitor binding could alleviate OP inhibition, the model was used to predict optimal activation coefficients to rescue severely inhibited NMJs. Considering this worst-case situation in which the majority of AChE would be inhibited, the inhibitor to activator ratio (IAR) at which the e.p.p. duration was 25% that of the uninhibited duration (Table 1) for several OP inhibitors was found, and used with a fully inhibited enzyme (inhibitor to enzyme ratio of 2:1) to calculate the lowest *ε-inh* for again returning the e.p.p. duration to within 25% of the uninhibited value. We hypothesize that the activator used here is allosterically activating, in that we observe changes in OP binding as being more critical than altering active site chemistry. Allosteric binding sites can be associated with long-range structural rearrangements [22] which could modulate the AChE active site cleft and “neck” without significantly altering the arrangement of the catalytic triad.

**Table 1:** **Predicted therapeutic parameters for preventing OP-induced intoxication**

***Inhibitor***	***Highest IAR*** ^***a***^	***Target e.p.p. duration***	***ε-inh*** ^***b***^
VX	1.43	0.939 s	.012
VR	1.67	2.604 s	.026
GA	10	0.639 s	.086
GB	1.67	0.627 s	.046
GD	5	1.704 s	.066
GF	3.33	2.604 s	.034
DFP	2	0.517 s	.094
Paraoxon	1.67	0.567 s	.080

^a^Highest ratio to return e.p.p. shift duration to 75% of inhibited at 1:1 inhibitor:AChE (partial AChE inhibition). *ε-on, ε-off, ε-cat = 1; ε-inh = 0.1.*

^b^Lowest activation coefficient to e.p.p. duration to 20% of uninhibited at 1:1 GB:AChE (full AChE inhibition) and IAR.

By using an ABM, we take into account the dynamic nature of the activation event. In this case, the binding of a single activator agent to a single enzyme agent will transiently alter the behavior of that enzyme (for example, reducing the inhibitor’s binding affinity). With an ODE-based model, this small nuance of a single agent changing activity would be lost due to the averaging achieved by considering the populations of all enzyme agents. Within the context of this model, and the example just provided, a single activator binding to a single enzyme would account for a change of only 2% of the enzymes present, a negligible signal in the ODE yet one that we have shown to produce significant emergent behavior using the ABM.

## Conclusions

Our model supports the hypothesis that as a potential therapeutic route, allosteric AChE activators provide a novel and useful approach for treating OP intoxication. Although our model does not rule out other methods and mechanisms of action, we contend that isolation and targeting the most effective allosteric therapies could produce life-saving effects by partially ameliorating the deleterious actions of AChE inhibitor binding.

## Methods

**Figure 4:**
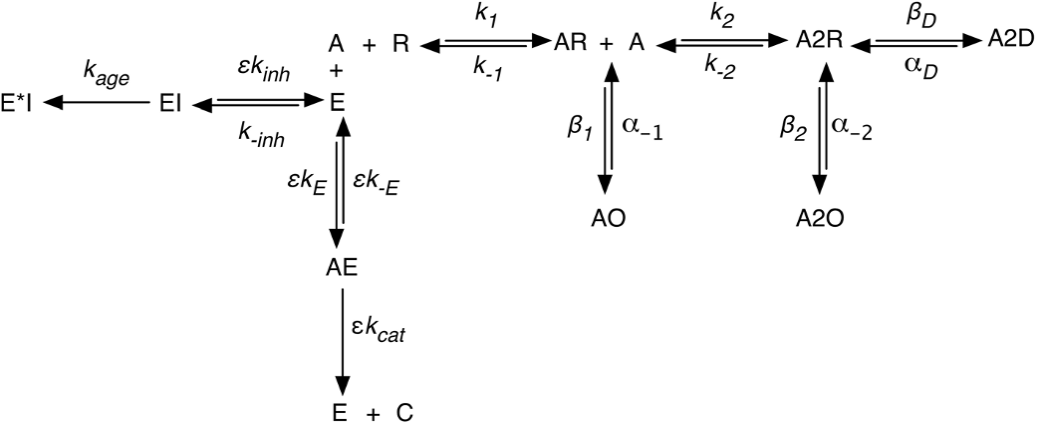
**Reaction diagram for nAChR-mediated signaling at the NMJ.**

Model development began with conceptually representative reaction diagrams, considering the binding of acetylcholine (ACh) to the nicotinic acetylcholine receptors (nAChR), ACh turnover by AChE, inhibition of AChE, and activation of AChE. These events can be represented by Figure 4, where A is ACh; R is the receptor; AR and AO are monoliganded closed and open receptors, respectively; A2R and A2O are diliganded closed and open receptors, respectively; A2D is the desensitized receptor; E is AChE; I is the inhibitor; and ε are the activation coefficients for substrate binding (*ε-on* and *ε-off*), catalysis (*ε-cat*), and inhibitor binding (*ε-inh*).

The system was modeled in NetLogo [23] as a single quantal injection of substrate [24]. The rules assigned to the individual agents are summarized below:

*nAChR*: cannot move; can bind one or two ACh molecules; can convert from closed and bound to open and bound with either one or two ACh ligands; can convert from open with two ligands to desensitized with two ligands; can release either ligand

*AChE*: cannot move; can bind single ACh molecule; can convert ACh to product; can bind inhibitor; can be aged by inhibitor; can bind activator; can release activator or ACh

*ACh*: can move with diffusion; can bind to nAChR or AChE; can be converted to product

*Inhibitor*: can move with diffusion; can bind to AChE; can induce AChE aging

*Activator*: can move with diffusion; can bind to AChE; can alter AChE activities

*Product*: disappears upon formation; increases accounting tracker by 1 unit

All of the microscopic rate constants were obtained directly from experimental reports, and there was no fitting of the model to observed data. The behavior resulting from each simulation run therefore emerges from the biophysical properties of the system and the definition of the rules. Receptor binding rates were parameterized from Hatton [25]. Enzyme turnover rates were obtained from Salih [26] and Hasinoff [27]. Inhibitor parameters were obtained from Hodge [28], Radic [29] and Worek [30] (Table 2).

**Table 2:** **Inhibitor parameters for model**

***Inhibitor***	***k-inh (M*** ^***-1***^ ***min*** ^***-1***^ ***)***	***k-age (h*** ^***-1***^ ***)***
***GA***	7.4 * 10 ^*6*^	*0.036*
***GB***	2.7* 10 ^*7*^	*0.228*
***GD***	9.2* 10 ^*7*^	*6.6*
***GF***	4.9* 10 ^*8*^	*0.099*
***VX***	1.2* 10 ^*8*^	*0.019*
***VR***	4.4* 10 ^*8*^	*0.005*
***Paraoxon***	1.2* 10 ^*6*^	*0.186*
***DFP***	1.3* 10 ^*5*^	*0.221*

### Supporting information available

The complete NetLogo code is included as supplemental information and is also available at the NetLogo model library (http://ccl.northwestern.edu/netlogo/models/community/index.cgi) Additional file 1.

## Authors’ information

RRC is a protein biochemist, and an expert in enzyme inhibition. PJR is a biological modeler with extensive experience in ODE modeling in advanced biology, physics and mathematics. JJS is a board-certified toxicologist, a medicinal chemist, and expert in acetylcholinesterase inhibition by OPs. JMG is a board-certified toxicologist, a biological modeler, and expert in the toxicology and modeling of OP pesticides and nerve agents.

## Electronic supplementary material

Additional file 1: **Supporting Information Legends.** NetLogo Code. Text code for the model. This code functions, but does not include the graphical user interface for the model, which is available at the NetLogo model library. (DOCX 35 KB)

## References

[1] Singh SP, Aggarwal AD, Oberoi SS, Aggarwal KK, Thind AS, Bhullar DS, Walia DS, Chahal PS (2013). Study of poisoning trends in north India – a perspective in relation to world statistics. J Forensic Leg Med.

[2] Jett DA, Yeung DT (2010). The CounterACT Research Network: basic mechanisms and practical applications. Proc Am Thorac Soc.

[3] Gupta B, Sharma R, Singh N, Kuca K, Acharya JR, Ghosh KK (2014). In vitro reactivation kinetics of paraoxon- and DFP-inhibited electric eel AChE using mono- and bis-pyridinium oximes. Arch Toxicol.

[4] Rosenberg YJ, Gearhart J, Mao L, Jiang X, Hernandez-Abanto S (2014). Protection against paraoxon toxicity by an intravenous pretreatment with polyethylene-glycol-conjugated recombinant butyrylcholinesterase in macaques. Chem Biol Interact.

[5] Nachon F, Brazzolotto X, Trovaslet M, Masson P (2013). Progress in the development of enzyme-based nerve agent bioscavengers. Chem Biol Interact.

[6] Abbas R, Hayton WL (1997). A physiologically based pharmacokinetic and pharmacodynamic model for paraoxon in rainbow trout. Toxicol Appl Pharmacol.

[7] Gearhart JM, Jepson GW, Clewell HJ, Andersen ME, Connolly RB (1994). Physiologically based pharmacokinetic model for the inhibition of acetylcholinesterase by organophosphate esters. Environ Health Perspect.

[8] Poet TS, Kousba AA, Dennison SL, Timchalk C (2004). Physiologically based pharmacokinetic/pharmacodynamic model for the organophosphorous pesticide diazinon. Neurotoxicology.

[9] Timchalk C, Poet TS (2008). Development of a physiologically based pharmacokinetic and pharmacodynamic model to determine dosimetry and cholinesterase inhibition for a binary mixture of chlorpyrifos and diazinon in the rat. Neurotoxicology.

[10] Gearhart JM, Jepson GW, Clewell HJ, Andersen ME, Conolly RB (1990). Physiologically based pharmacokinetic and pharmacodynamic model for the inhibition of acetylcholinesterase by diisopropylfluorophosphate. Toxicol Appl Pharmacol.

[11] Chen K, Seng KY (2012). Calibration and validation of a physiologically based model for soman intoxication in the rat, marmoset, guinea pig and pig. J Appl Toxicol.

[12] Van der Merwe D, Brooks JD, Gehring R, Baynes RE, Monteiro-Riviere NA, Rivierre JE (2006). A physiologically based pharmacokinetic model of organophosphate dermal absorption. Toxicol Sci.

[13] Bonabeau E (2002). Agent-based modeling: methods and techniques for simulating human systems. Proc Natl Acad Sci USA.

[14] An G, Mi Q, Dutta-Moscato J, Vodovotz Y (2009). Agent-based models in translational systems biology. Wiley Interdiscip Rev Syst Biol Med.

[15] Pogson M, Holcomb M, Smallwood R, Qwarnstrom E (2008). Introducing spatial information into predictive NF-kB modelling – an agent-based approach. PLoS ONE.

[16] An G (2009). A model of TLR4 signaling and tolerance using a qualitative, particle-event-based method: introduction of spatially configured stochastic reaction chambers (SCSRC). Math Biosci.

[17] Zhang L, Athale CD, Deisboeck TS (2007). Development of a three-dimensional multiscale agent-based tumor model: simulating gene-protein interaction profiles, cell phenotypes and multicellular patterns in brain cancer. J Theor Biol.

[18] Kaul H, Ventikos Y (2013). Investigating biocomplexity through the agent-based paradigm. Brief Bioinform.

[19] Lipniacki T, Puszynski K, Paszek P, Brasier AR, Kimmel M (2007). Single TNFalpha trimmers mediating NF-kappaB activation: stochastic robustness of NF-kappaB signaling. BMC Bioinform.

[20] Katz B, Miledi R (1973). The binding of acetylcholine to receptors and its removal from the synaptic cleft. J Physiol.

[21] Kordas M (1977). On the role of junctional cholinesterase in determining the time course of the end-plate current. J Physiol.

[22] Sagermann M, Chapleau RR, DeLorimier E, Lei M (2009). Using affinity chromatography to engineer and characterize pH-dependent protein switches. Prot Sci.

[23] Wilenski U (1997). NetLogo.

[24] Land BR, Salpeter EE, Salpeter MM (1980). Acetylcholine receptor site density affects the rising phase of miniature endplate currents. Proc Natl Acad Sci USA.

[25] Hatton CJ, Shelley C, Brydson M, Beeson D, Colquhoun D (2003). Properties of the human muscle nicotinic receptor, and of the slow-channel myasthenic syndrome mutant εL221F, inferred from maximum likelihood fits. J Physiol.

[26] Salih E (1992). Catalysis by acetylcholinesterase in two-hydronic-reactive states: integrity of deuterium oxide effects and hydron inventories. Biochem J.

[27] Hasinoff BB (1982). Kinetics of acetylthiocholine binding to electric eel acetylcholinesterase in glycerol/water solvents of increased viscosity: Evidence for a diffusion-controlled reaction. Biochim Biophys Acta.

[28] Hodge AS, Humphrey DR, Rosenberry TL (1992). Ambenonium is a rapidly reversible noncovalent inhibitor of acetylcholinesterase, with one of the highest known affinities. Mol Pharmacol.

[29] Radic Z, Taylor P (2001). Interaction kinetics of reversible inhibitors and substrates with acetylcholinesterase and its fasciculin 2 complex. J Biol Chem.

[30] Worek F, Thiermann H, Szinicz L, Eyer P (2004). Kinetic analysis of interactions between human acetylcholinesterase, structurally different organophosphorus compounds and oximes. Biochem Pharmacol.

