# Supplementary material for: Potential new therapeutic modality revealed through agent-based modeling of the neuromuscular junction and acetylcholinesterase inhibition
Source: Theor Biol Med Model. 2014 Oct 2;11:42. doi: 10.1186/1742-4682-11-42 (PMC4209019; doi:10.1186/1742-4682-11-42)
Supplement: Supplementary file 1 — Additional file 1: Supporting Information Legends. NetLogo Code. Text code for the model. This code functions, but does not include the graphical user interface for the model, which is available at the NetLogo model library. (DOCX 35 KB) [file 12976_2014_483_MOESM1_ESM.docx]

**Agent-based modeling of the neuromuscular junction reveals insights into acetylcholinesterase inhibition and potential therapeutic modalities**

Richard R Chapleau, Peter J Robinson, John J Schlager, and Jeffery M Gearhart

**Supplemental Information**

NetLogo Code

;;;;;;;;;;;;;;;;;;;;;;;;;;;;;;;;;;;;;;;;;;;;;;;;;;;;;;;;;;;;;;;;;;;;;;

;;;;;;;;;;;;;;;;;;;;;;;;;;;;;;;;;;;;;;;;;;;;;;;;;;;;;;;;;;;;;;;;;;;;;;

;;;;;;;;;;;;;;;;;;;;;;;; GLOBAL VARIABLES ;;;;;;;;;;;;;;;;;;;;;;;;;;;;

;;;;;;;;;;;;;;;;;;;;;;;;;;;;;;;;;;;;;;;;;;;;;;;;;;;;;;;;;;;;;;;;;;;;;;

;;;;;;;;;;;;;;;;;;;;;;;;;;;;;;;;;;;;;;;;;;;;;;;;;;;;;;;;;;;;;;;;;;;;;;

globals [

;;;;;;;;;;;;;;;;;;;;;;;;;;;;;;;;;;;;;;;;;;;;;;;;;;;;;;;;;;;;;;;;;;;;;;

;;;;;;;;;;;;; Nicotinic Acetylcholine receptors ;;;;;;;;;;;;;;;;;;;;;;

;;;;;;;;;;;;;;;;;;;;;;;;;;;;;;;;;;;;;;;;;;;;;;;;;;;;;;;;;;;;;;;;;;;;;;

k1 ; association rate at first site

k-1 ; dissociation rate of first site

k2 ; association rate at second site

k-2 ; dissociation rate of second site

b1 ; opening rate with one ligand

a1 ; closing rate with one ligand

b2 ; opening rate with two ligands

a2 ; closing rate with two ligands

bD ; desensitization rate with two ligands

aD ; resensitization rate with two ligands

;;;;;;;;;;;;;;;;;;;;;;;;;;;;;;;;;;;;;;;;;;;;;;;;;;;;;;;;;;;;;;;;;;;;;;

;;;;;;;;;;;;; Acetylcholinesterase ;;;;;;;;;;;;;;;;;;;;;;;;;;;;;;;;;;;

;;;;;;;;;;;;;;;;;;;;;;;;;;;;;;;;;;;;;;;;;;;;;;;;;;;;;;;;;;;;;;;;;;;;;;

ke ; association rate of substrate

k-e ; dissociation rate of substrate

kcat ; catalytic turnover rate

kinh ; inhibitor on-rate

k-inh ; inhibitor off-rate

kage ; inhibitor aging constant

kact ; activator on-rate

k-act ; activator off-rate

eps-on ; activation coefficient for substrate binding

eps-off ; activation coefficient for substrate release

eps-cat ; activation coefficient for enzyme catalysis

eps-inh ; activation coefficient for inhibitor binding

;;;;;;;;;;;;;;;;;;;;;;;;;;;;;;;;;;;;;;;;;;;;;;;;;;;;;;;;;;;;;;;;;;;;;;

;;;;;;;;;;;;; Agent Accounting ;;;;;;;;;;;;;;;;;;;;;;;;;;;;;;;;;;;;;;;

;;;;;;;;;;;;;;;;;;;;;;;;;;;;;;;;;;;;;;;;;;;;;;;;;;;;;;;;;;;;;;;;;;;;;;

sumOpen ; total number of open NARs

sumC ; total number of Cholines produced

nC ; number of choline

nA ; number of ACh

nI ; number of inhibitors

nAct ; number of activators

;;;;;;;;;;;;;;;;;;;;;;;;;;;;;;;;;;;;;;;;;;;;;;;;;;;;;;;;;;;;;;;;;;;;;;

;;;;;;;;;;;;; Run Control ;;;;;;;;;;;;;;;;;;;;;;;;;;;;;;;;;;;;;;;;;;;;

;;;;;;;;;;;;;;;;;;;;;;;;;;;;;;;;;;;;;;;;;;;;;;;;;;;;;;;;;;;;;;;;;;;;;;

dt ; time step

dtp ; time step-print

tend ; end of simulation time

time ; time

;;;;;;;;;;;;;;;;;;;;;;;;;;;;;;;;;;;;;;;;;;;;;;;;;;;;;;;;;;;;;;;;;;;;;;

;;;;;;;;;;;;; Environmental Settings ;;;;;;;;;;;;;;;;;;;;;;;;;;;;;;;;;

;;;;;;;;;;;;;;;;;;;;;;;;;;;;;;;;;;;;;;;;;;;;;;;;;;;;;;;;;;;;;;;;;;;;;;

Vol ; volume (nm3)

As0 ; initial amount of ACh

Ain ; amount of ACh per injection

Diff ; diffusion coeffecient (nm2/s)

]

;;;;;;;;;;;;;;;;;;;;;;;;;;;;;;;;;;;;;;;;;;;;;;;;;;;;;;;;;;;;;;;;;;;;;;

;;;;;;;;;;;;;;;;;;;;;;;;;;;;;;;;;;;;;;;;;;;;;;;;;;;;;;;;;;;;;;;;;;;;;;

;;;;;;;;;;;;;;;;;;;;;;;; BREED DEFINITIONS ;;;;;;;;;;;;;;;;;;;;;;;;;;;

;;;;;;;;;;;;;;;;;;;;;;;;;;;;;;;;;;;;;;;;;;;;;;;;;;;;;;;;;;;;;;;;;;;;;;

;;;;;;;;;;;;;;;;;;;;;;;;;;;;;;;;;;;;;;;;;;;;;;;;;;;;;;;;;;;;;;;;;;;;;;

breed [ Rs R ] ; unbound NARs

breed [ ARs AR ] ; closed monoliganded NARs

breed [ A2Rs A2R ] ; closed diliganded NARs

breed [ AOs AO ] ; open monoliganded NARs

breed [ A2Os A2O ] ; open diliganded NARs

breed [ A2Ds A2D ] ; desensitized NARs

breed [ tEns tEn ] ; unbound pre-synaptic AChE

breed [ AtEs AtE ] ; bound pre-synaptic AChE

breed [ As A ] ; free ACh

breed [ Cs C ] ; free choline

breed [ bEns bEn ] ; unbound post-synaptic AChE

breed [ AbEs AbE ] ; bound post-synaptic AChE

breed [ A-ins A-in ] ; new ACh for signal injections

breed [ Inhs Inh ] ; inhibitors

breed [ ItEs ItE ] ; inhibited pre-synaptic enyzme

breed [ IbEs IbE ] ; inhibited post-synaptic enzyme

breed [ Ageds Aged ] ; aged enzymes

breed [ Acts Act ] ; activators

breed [ ActEs ActE ] ; activated pre-synaptic enzyme

breed [ AcbEs AcbE ] ; activated post-synaptic enzyme

breed [ AcAtEs AcAtE ] ; activated, substrate bound pre-syn enzyme

breed [ AcAbEs AcAbE ] ; activated, substrate bound post-syn enzyme

breed [ AcItEs AcItE ] ; activated, pre-synaptic inhibited enzyme

breed [ AcIbEs AcIbE ] ; activated, post-synaptic enzyme

;;;;;;;;;;;;; Breed Variable ;;;;;;;;;;;;;;;;;;;;;;;;;;;;;;;;;;;;;;;;;

turtles-own [ partner ]

;;;;;;;;;;;;;;;;;;;;;;;;;;;;;;;;;;;;;;;;;;;;;;;;;;;;;;;;;;;;;;;;;;;;;;

;;;;;;;;;;;;;;;;;;;;;;;;;;;;;;;;;;;;;;;;;;;;;;;;;;;;;;;;;;;;;;;;;;;;;;

;;;;;;;;;;;;;;;;;;;;;;;; SETUP ;;;;;;;;;;;;;;;;;;;;;;;;;;;;;;;;;;;;;;;

;;;;;;;;;;;;;;;;;;;;;;;;;;;;;;;;;;;;;;;;;;;;;;;;;;;;;;;;;;;;;;;;;;;;;;

;;;;;;;;;;;;;;;;;;;;;;;;;;;;;;;;;;;;;;;;;;;;;;;;;;;;;;;;;;;;;;;;;;;;;;

To setup

clear-all

reset-ticks

random-seed 1

;;; Environmental Control ;;;

set dt 0.0005

set dtp 1

set tend 1

set time 0

set Vol 50000

set Diff 4e8

;;; Configure Activators and Inhibitors ;;;

if (inhibitor = "None") [

set kinh 0

set kage 0

set Inhibitors 0 ]

if (inhibitor = "VX") [

set kinh 1.2e8 * 0.276763 ; converts L/mol*min to nm3/molecule*s

set kage 0.019 * 2.7778e-4 ] ; converts h-1 to s-1

if (inhibitor = "VR") [

set kinh 4.4e8 * 0.276763

set kage 0.005 * 2.7778e-4 ]

if (inhibitor = "GA") [

set kinh 7.4e6 * 0.276763

set kage .036 * 2.7778e-4 ]

if (inhibitor = "GB") [

set kinh 2.7e7 * 0.276763

set kage 0.228 * 2.7778e-4 ]

if (inhibitor = "GD") [

set kinh 9.2e7 * 0.276763

set kage 6.6 * 2.7778e-4 ]

if (inhibitor = "GF") [

set kinh 4.9e8 * 0.276763

set kage 0.099 * 2.7778e-4 ]

if (inhibitor = "Paraoxon") [

set kinh 1.2e6 * 0.276763

set kage 0.186 * 2.7778e-4 ]

if (inhibitor = "DFP") [

set kinh 1.3e5 * 0.276763

set kage 0.221 * 2.7778e-4 ]

if (activator = "None") [

set kact 0

set k-act 0

set eps-on 1

set eps-off 1

set eps-cat 1

set eps-inh 1

set Activators 0 ]

if (activator = "Activator") [

; These coefficients can be changed to evaluate effects of activation

set kact 1e7

set k-act 1

set eps-on 1

set eps-off 1

set eps-cat 1

set eps-inh 1

]

;;; Agent Accounting Parameters ;;;

set sumOpen 0

set sumC 0

set nA 0

set nC 0

set nI Inhibitors

set nAct Activators

;;; Receptor Parameters ;;;

set k1 2.23e7 * 1.660578 ; converts L/mol*s to nm3/molecule*s

set k-1 1330

set k2 4.7e8 * 1.660578 ; converts L/mol*s to nm3/molecule*s

set k-2 13400

set b1 38

set a1 6000

set b2 51600

set a2 2460

set bD 65.2

set aD 775

;;; Enzyme Parameters ;;;

set ke 2.1e8 * 1.660578

set k-e 1.9e4

set kcat 6.8e3

;;; Agent Creation ;;;

set As0 200

set Ain 200

add-inhibitors

add-activators

create-Rs 200

create-tEns 25

create-bEns 25

create-As As0

ask Rs [ setxy random-xcor 0 ]

ask tEns [ setxy random-xcor 49 ]

ask bEns [ setxy random-xcor 0 ]

ask As [ setxy random-xcor 49 ]

ask turtles [ set partner nobody ]

end

;;;;;;;;;;;;;;;;;;;;;;;;;;;;;;;;;;;;;;;;;;;;;;;;;;;;;;;;;;;;;;;;;;;;;;

;;;;;;;;;;;;;;;;;;;;;;;;;;;;;;;;;;;;;;;;;;;;;;;;;;;;;;;;;;;;;;;;;;;;;;

;;;;;;;;;;;;;;;;;;;;;;;; RUNNING FUNCTIONS ;;;;;;;;;;;;;;;;;;;;;;;;;;;

;;;;;;;;;;;;;;;;;;;;;;;;;;;;;;;;;;;;;;;;;;;;;;;;;;;;;;;;;;;;;;;;;;;;;;

;;;;;;;;;;;;;;;;;;;;;;;;;;;;;;;;;;;;;;;;;;;;;;;;;;;;;;;;;;;;;;;;;;;;;;

;;;;;;;;;;;;; Main Go Loop ;;;;;;;;;;;;;;;;;;;;;;;;;;;;;;;;;;;;;;;;;;;

To go

;;; Calculate Accounting Variables ;;;

set time (time + dt)

set sumC (sumC + (count (Cs)))

set nA count ( As )

set sumOpen (count (AOs) + count (A2Os))

set sumAllOpen (sumAllOpen + sumOpen)

;;; Partner removal ;;;

ask turtles [ set partner nobody ]

;;; Execute commands ;;;

ask Cs [die]

ask Inhs [ move ]

ask Acts [ move ]

ask As [ move ]

ask Inhs [ inhibitor-complex ]

ask Acts [ activator-complex ]

ask As [ substrate-complex ]

ask A2Rs [ closed ]

ask ARs [ closed ]

ask A2Os [ open ]

ask AOs [ open ]

ask AtEs [ turnover ]

ask AbEs [ turnover ]

ask AcAtEs [ turnover ]

ask AcAbEs [ turnover ]

ask ItEs [ age ]

ask IbEs [ age ]

ask AcItEs [ age ]

ask AcIbEs [ age ]

;;; Iteration ending ;;;

tick

if time > tend [ stop ]

end

;;;;;;;;;;;;;;;;;;;;;;;;;;;;;;;;;;;;;;;;;;;;;;;;;;;;;;;;;;;;;;;;;;;;;;

;;;;;;;;;;;;; Substrate Procedures ;;;;;;;;;;;;;;;;;;;;;;;;;;;;;;;;;;;

;;;;;;;;;;;;;;;;;;;;;;;;;;;;;;;;;;;;;;;;;;;;;;;;;;;;;;;;;;;;;;;;;;;;;;

;;;;;;;;;;;;; Substrate Move Loop ;;;;;;;;;;;;;;;;;;;;;;;;;;;;;;;;;;;;

to move

fd dt * Diff

rt dt * Diff

end

;;;;;;;;;;;;; Substrate Binding Loop ;;;;;;;;;;;;;;;;;;;;;;;;;;;;;;;;;

to substrate-complex

if partner != nobody [ stop ]

set partner one-of other turtles-here with [breed != As and partner =

nobody]

if partner = nobody [ stop ]

if [partner] of partner != nobody [ stop ]

ifelse ((partner != nobody and ([breed] of partner) = Rs) and ((random- float 1) < ((k1 * nA / Vol) / (((k1 * nA / Vol) + k-1)))))

[ set (breed) ARs

ask partner [die]

set partner nobody ]

[ ifelse ((partner != nobody and ([breed] of partner) = ARs) and

((random-float 1) < ((k2 * nA / Vol) / (((k2 * nA / Vol) + k-

2)))))

[ set (breed) A2Rs

ask partner [die]

set partner nobody ]

[ ifelse ((partner != nobody and ([breed] of partner) = tEns) and

((random-float 1) < ((ke * nA / Vol) / (((ke * nA / Vol) +

k-e)))))

[ set (breed) AtEs

ask partner [die]

set partner nobody ]

[ ifelse ((partner != nobody and ([breed] of partner) = bEns) and

((random-float 1) < ((ke * nA / Vol) / (((ke * nA / Vol) +

k-e)))))

[ set (breed) AbEs

ask partner [die]

set partner nobody ]

[ ifelse ((partner != nobody and ([breed] of partner) = ActEs)

and ((random-float 1) < ((eps-on * ke * nA / Vol) /

((eps-on * ke * nA / Vol) + (eps-off * k-e)))))

[ set (breed) AcAtEs

ask partner [die]

set partner nobody ]

[ ifelse ((partner != nobody and ([breed] of partner) = AcbEs)

and ((random-float 1) < ((eps-on * ke * nA / Vol) /

((eps-on * ke * nA / Vol) + (eps-off * k-e)))))

[ set (breed) AcAbEs

ask partner [die]

set partner nobody ]

[ ask partner [ set partner nobody ]

set partner nobody ] ] ] ] ] ]

end

;;;;;;;;;;;;;;;;;;;;;;;;;;;;;;;;;;;;;;;;;;;;;;;;;;;;;;;;;;;;;;;;;;;;;;

;;;;;;;;;;;;; Receptor Procedures ;;;;;;;;;;;;;;;;;;;;;;;;;;;;;;;;;;;;

;;;;;;;;;;;;;;;;;;;;;;;;;;;;;;;;;;;;;;;;;;;;;;;;;;;;;;;;;;;;;;;;;;;;;;

;;;;;;;;;;;;; Closed Receptor Loop ;;;;;;;;;;;;;;;;;;;;;;;;;;;;;;;;;;;

to closed

ifelse breed = A2Rs

[ ifelse ((random-float 1) < (dt * bD / (dt * (bD + aD + k-2 + b2 +

a2 + (nA * k2 / Vol)))))

[ set (breed) A2Ds ]

[ ifelse ((random-float 1) < (dt * k-2 / (dt * (bD + aD + k-2 +

b2 + a2 + (nA * k2 / Vol)))))

[ set (breed) ARs

hatch-As 1 ]

[ ifelse ((random-float 1) < (dt * k1 / (dt * (bD + aD + k-2 +

b2 + a2 + (nA * k2 / Vol)))))

[ set (breed) A2Os ]

[ stop ] ] ] ]

[ if breed = ARs

[ ifelse ((random-float 1) < (dt * b1 / (dt * (a1 + b1 + k-1 +

(nA * k1 / Vol)))))

[ set (breed) AOs ]

[ ifelse ((random-float 1) < (dt * k-1 / (dt * (a1 + b1 + k-1 +

(nA * k1 / Vol)))))

[ set (breed) Rs

hatch-As 1 ]

[ stop ] ] ] ]

end

;;;;;;;;;;;;; Open Receptor Loop ;;;;;;;;;;;;;;;;;;;;;;;;;;;;;;;;;;;;;

to open

ifelse breed = A2Os

[ ifelse ((random-float 1) < (a2 / (a2 + b2)))

[ set (breed) A2Rs ]

[ stop ] ]

[ if breed = AOs

[ ifelse ((random-float 1) < (a1 / (a1 + b1)))

[ set (breed) ARs ]

[ stop ] ] ]

end

;;;;;;;;;;;;;;;;;;;;;;;;;;;;;;;;;;;;;;;;;;;;;;;;;;;;;;;;;;;;;;;;;;;;;;

;;;;;;;;;;;;; Enzyme Procedures ;;;;;;;;;;;;;;;;;;;;;;;;;;;;;;;;;;;;;;

;;;;;;;;;;;;;;;;;;;;;;;;;;;;;;;;;;;;;;;;;;;;;;;;;;;;;;;;;;;;;;;;;;;;;;

;;;;;;;;;;;;; Enzyme Turnover Loop ;;;;;;;;;;;;;;;;;;;;;;;;;;;;;;;;;;;

to turnover

ifelse breed = AtEs

[ ifelse (((dt * kcat) / (dt * (k-e + kcat))) > (random-float 1))

[ set (breed) tEns

set partner nobody

hatch-Cs 1 ]

[ stop ] ]

[ ifelse breed = AbEs

[ ifelse (((dt * kcat) / (dt * (k-e + kcat))) > (random-float 1))

[ set (breed) bEns

set partner nobody

hatch-Cs 1 ]

[ stop ] ]

[ ifelse breed = AcAtEs

[ ifelse (((dt * eps-cat * kcat) / (dt * ((eps-off * k-e) +

(eps-cat * kcat)))) > (random-float 1))

[ set (breed) ActEs

set partner nobody

hatch-Cs 1 ]

[ stop ]]

[ ifelse breed = AcAbEs

[ if (((dt * eps-cat * kcat) / (dt * ((eps-off * k-e) +

(eps-cat * kcat)))) > (random-float 1))

[ set (breed) AcbEs

set partner nobody

hatch-Cs 1 ] ]

[ stop ] ] ] ]

end

;;;;;;;;;;;;; Enzyme Aging Loop ;;;;;;;;;;;;;;;;;;;;;;;;;;;;;;;;;;;;;;

to age

ifelse breed = ItEs

[ ifelse (((dt * kage) / (dt * (k-inh + (kinh * nI / Vol) + kage))) >

(random-float 1))

[ set (breed) Ageds

set partner nobody ]

[ if ((random-float 1) < ((dt * k-inh) / (dt * (k-inh + (kinh * Ni

/ Vol) + kage))))

[ set (breed) tEns

set partner nobody

hatch-Inhs 1 ] ] ]

[ ifelse (((dt * kage) / (dt * (k-inh + (kinh * nI / Vol) + kage))) >

(random-float 1))

[ set (breed) Ageds

set partner nobody ]

[ if ((random-float 1) < ((dt * k-inh) / (dt * (k-inh + (kinh * nI

/ Vol) + kage))))

[ set (breed) bEns

set partner nobody

hatch-Inhs 1 ] ] ]

end

;;;;;;;;;;;;;;;;;;;;;;;;;;;;;;;;;;;;;;;;;;;;;;;;;;;;;;;;;;;;;;;;;;;;;;

;;;;;;;;;;;;; Inhibitor Procedures ;;;;;;;;;;;;;;;;;;;;;;;;;;;;;;;;;;;

;;;;;;;;;;;;;;;;;;;;;;;;;;;;;;;;;;;;;;;;;;;;;;;;;;;;;;;;;;;;;;;;;;;;;;

;;;;;;;;;;;;; Inhibitor Addition Loop ;;;;;;;;;;;;;;;;;;;;;;;;;;;;;;;;

to add-inhibitors

create-Inhs Inhibitors

ask Inhs

[ setxy random-xcor random-ycor

set partner nobody ]

end

;;;;;;;;;;;;; Enzyme Inhibition Loop ;;;;;;;;;;;;;;;;;;;;;;;;;;;;;;;;;

to inhibitor-complex

if partner != nobody [ stop ]

set partner one-of (other turtles-here with [breed != As])

if partner = nobody [ stop ]

ifelse ((partner != nobody and ([breed] of partner) = tEns) and

((random-float 1) < ((kinh * nI / Vol) / ((kinh * nI / Vol) +

1))))

[ set (breed) ItEs

ask partner [die]

set partner nobody ]

[ ifelse ((partner != nobody and ([breed] of partner) = bEns) and

((random-float 1) < ((kinh * nI / Vol) / ((kinh * nI / Vol) +

1))))

[ set (breed) IbEs

ask partner [die]

set partner nobody ]

[ ifelse ((partner != nobody and ([breed] of partner) = ActEs) and

((random-float 1) < ((eps-inh * kinh * nI / Vol) / ((eps-

inh * kinh * nI / Vol) + 1))))

[ set (breed) AcItEs

ask partner [die]

set partner nobody ]

[ ifelse ((partner != nobody and ([breed] of partner) = AcbEs)

and ((random-float 1) < ((eps-inh * kinh * nI / Vol) /

((eps-inh * kinh * nI / Vol) + 1))))

[ set (breed) AcIbEs

ask partner [die]

set partner nobody ]

[ ask partner [ set partner nobody ]

set partner nobody ] ] ] ]

end

;;;;;;;;;;;;;;;;;;;;;;;;;;;;;;;;;;;;;;;;;;;;;;;;;;;;;;;;;;;;;;;;;;;;;;

;;;;;;;;;;;;; Activator Procedures ;;;;;;;;;;;;;;;;;;;;;;;;;;;;;;;;;;;

;;;;;;;;;;;;;;;;;;;;;;;;;;;;;;;;;;;;;;;;;;;;;;;;;;;;;;;;;;;;;;;;;;;;;;

;;;;;;;;;;;;; Activator Additon Loop ;;;;;;;;;;;;;;;;;;;;;;;;;;;;;;;;;

to add-activators

create-Acts Activators

ask Acts

[ setxy random-xcor random-ycor

set partner nobody ]

end

;;;;;;;;;;;;; Enzyme Activation Loop ;;;;;;;;;;;;;;;;;;;;;;;;;;;;;;;;;

to activator-complex

if partner != nobody [ stop ]

set partner one-of (other turtles-here with [breed != As])

if partner = nobody [ stop ]

if ([breed] of partner = As)

[ ask partner [ set partner nobody] set partner nobody stop ]

ifelse ((partner != nobody and ([breed] of partner) = tEns) and

((random-float 1) < ((dt * kact * nAct / Vol) / (dt * ((kact *

nAct / Vol) + k-act)))))

[ set (breed) ActEs

ask partner [ die ]

set partner nobody ]

[ ifelse ((([breed] of partner) = bEns) and ((random-float 1) < ((dt

* kact * nAct / Vol) / (dt * ((kact * nAct / Vol) +

k-act)))))

[ set (breed) AcbEs

ask partner [ die ]

set partner nobody ]

[ ask partner [ set partner nobody ]

set partner nobody ] ]

end
